# HCMV Reprogramming of Infected Monocyte Survival and Differentiation: A Goldilocks Phenomenon

**DOI:** 10.3390/v6020782

**Published:** 2014-02-13

**Authors:** Emily V. Stevenson, Donna Collins-McMillen, Jung Heon Kim, Stephen J. Cieply, Gretchen L. Bentz, Andrew D. Yurochko

**Affiliations:** 1 Department of Microbiology and Immunology, Louisiana State University Health Sciences Center-Shreveport, Shreveport, LA 71130, USA; E-Mails: esteve@lsuhsc.edu (E.V.S.); dcoll2@lsuhsc.edu (D.C.-M.); jkim4@lsuhsc.edu (J.H.K.); sciepl@lsuhsc.edu (S.J.C.); Bentz_GL@Mercer.edu (G.L.B.); 2 Center for Molecular and Tumor Virology, Louisiana State University Health Sciences Center-Shreveport, Shreveport, LA 71130, USA; 3 Feist-Weiller Cancer Center, Louisiana State University Health Sciences Center-Shreveport, Shreveport, LA 71130, USA

**Keywords:** HCMV, monocyte, macrophage, polarization, survival, signaling, receptor-ligand, viral persistence, viral dissemination

## Abstract

The wide range of disease pathologies seen in multiple organ sites associated with human cytomegalovirus (HCMV) infection results from the systemic hematogenous dissemination of the virus, which is mediated predominately by infected monocytes. In addition to their role in viral spread, infected monocytes are also known to play a key role in viral latency and life-long persistence. However, in order to utilize infected monocytes for viral spread and persistence, HCMV must overcome a number of monocyte biological hurdles, including their naturally short lifespan and their inability to support viral gene expression and replication. Our laboratory has shown that HCMV is able to manipulate the biology of infected monocytes in order to overcome these biological hurdles by inducing the survival and differentiation of infected monocytes into long-lived macrophages capable of supporting viral gene expression and replication. In this current review, we describe the unique aspects of how HCMV promotes monocyte survival and differentiation by inducing a “finely-tuned” macrophage cell type following infection. Specifically, we describe the induction of a uniquely polarized macrophage subset from infected monocytes, which we argue is the ideal cellular environment for the initiation of viral gene expression and replication and, ultimately, viral spread and persistence within the infected host.

## 1. Introduction

Human cytomegalovirus (HCMV), a ubiquitous betaherpesvirus that infects 60%–90% of the population worldwide, establishes a lifelong infection of the host [1]. The disease manifestations associated with HCMV infection are dependent upon the immune status of the host. In healthy individuals, primary infection with HCMV is typically asymptomatic, although mononucleosis-like symptoms have been reported [2,3]. Long-term infection with HCMV, however, is associated with chronic inflammation, which links HCMV infection to the development of cardiovascular diseases and some types of cancers in otherwise immunocompetent individuals [3,4,5]. In contrast, HCMV infection causes severe morbidity and mortality in immunocompromised individuals, including AIDS patients, transplant recipients, and developing fetuses [3,6]. In these susceptible populations lacking normal immune control of the virus, HCMV infection can lead to a severe disease state that is characterized by multi-organ system involvement [7]. 

The ability of HCMV to establish persistent infection within the host and the wide range of associated disease pathologies in multiple organ sites are a result of the systemic dissemination of the virus following primary infection. The dissemination strategy of HCMV involves a hematogenous step that is mediated predominately by infected peripheral blood monocytes. There are multiple lines of evidence supporting a specific role for monocytes in the hematogenous dissemination of HCMV. An HCMV cell-associated viremia is known to occur early after infection [8,9] and HCMV viral DNA is found predominately in peripheral blood monocytes and polymorphonuclear leukocytes (PMNs), rather than in lymphocytes [10,11,12]. Both monocytes and PMNs have been shown to play a role in the transfer of HCMV to blood vessel endothelial cells, suggesting a role for both cell types in the spread of HCMV [11]. Neither cell type is able to support viral replication [11]; however, viral gene expression and replication have been identified in monocyte-derived macrophages [13,14,15,16]. Furthermore, monocytes are the most prevalent infiltrating cell type found within HCMV-infected tissues [11,17], and a critical role for monocytes in the dissemination strategy of HCMV has been clearly defined in an animal model using the related murine CMV [18]. Based on these studies and others (reviewed in [19,20]), our laboratory focused on primary HCMV infection and the specific role monocytes play in viral dissemination [20,21]. We argue that monocytes serve as “Trojan Horses” to carry HCMV to multiple organ sites in the absence of *de novo* viral gene expression, as monocytes are initially non-permissive for viral gene expression and replication and only become permissive upon their differentiation into macrophages [20,21,22,23,24,25,26,27]. 

Monocytes/myeloid cells are not only key to viral spread following primary infection, but are central to the entire viral persistence strategy, as myeloid progenitors have been shown to be critical for the establishment of viral latency within the bone marrow [8,10,28,29,30]. Furthermore, reactivation of the virus occurs in these latently infected myeloid precursor cells that then leave the bone marrow as differentiated monocytes—thus also serving as a source of life-long periodic viral shedding [31,32,33]. Therefore, cells of the monocytic lineage play a defined and crucial role in the overall success of the virus in establishing a productive and persistent infection within the host and also in the transfer of HCMV to new hosts. Our recent studies have focused on determining how HCMV is able to manipulate the biology of newly infected monocytes during primary infection in order to promote the viral dissemination required for productive and persistent infection.

## 2. HCMV Rapidly Changes the Monocyte Cellular Environment Following Primary Infection to Establish a Pro-Viral Inflammatory Phenotype that Mediates Viral Dissemination

Due to the immune surveillance function of peripheral blood monocytes, this cell type can readily migrate through the blood vessel endothelium and infiltrate into peripheral organs, making infected monocytes ideal candidates for widespread delivery of the virus to nearly all organ tissues. Monocytes possess, however, a short lifespan of approximately 1–3 days within the bloodstream [34,35]. As previously mentioned, newly infected monocytes do not support *de novo* viral lytic gene expression or replication [36], thus new viral gene products are not synthesized that can alter the cellular environment to favor the success of the virus within the cell, as seen in other cell types infected by HCMV [37,38,39,40,41]. Despite this lack of new viral gene expression, our laboratory has observed rapid signaling and activation-induced changes in infected monocytes following viral binding that result in the production of a pro-inflammatory monocyte phenotype that favors viral spread [21,22,23,27,42,43,44]. Furthermore, we have shown that these phenotypic changes are mediated by the signaling that results from viral engagement of the epidermal growth factor receptor (EGFR) and the β_1_ and β_3_ integrins on the surface of monocytes [20,24,25,26,45,46]. Based on our recent studies [20,25,26,45,46] and the work of others [47,48,49], we have gained a better understanding of the early binding events that occur during HCMV infection of monocytes and how these events translate into the activation of cellular signaling pathways and functional changes in infected monocytes that promote the viral dissemination and persistence strategy [20,21].

During primary infection of monocytes, initial viral tethering likely occurs through engagement of glycoprotein M (gM)/gN to heparin sulfate proteoglycans [50]. This initial viral tethering event is then followed by the binding of the gH/gL/UL128-131 complex to specific proteinaceous receptors, the β_1_ and β_3_ integrins [26,46] and the binding of other viral glycoprotein(s), such as gB to EGFR on the surface of monocytes [45]. These receptor-binding events then trigger the activation of signaling cascades downstream of EGFR and integrins. Our data suggest that there are both distinct and overlapping signaling events that mediate viral entry into monocytes and that initiate specific monocyte biological changes [20,25,26,45,46]. For instance, the binding to and activation of both EGFR and integrins are required for viral entry into monocytes [26,45,46]. Moreover, the activation of both EGFR and integrins by HCMV is required for increased monocyte cellular motility, which is essential for the overall dissemination strategy of HCMV [26,45]. However, although both EGFR and integrin signaling are required for HCMV-induced enhanced monocyte motility, our data indicate that HCMV activation of EGFR alone promotes the upregulation of an actin cytoskeletal regulator, N‑WASP, which we showed to be required for enhanced monocyte motility [45]; while activation of integrins alone promotes the activation and upregulation of the actin cytoskeletal regulator, paxillin, which we show also induces monocyte motility and plays a key role in the viral entry process [26]. These data suggest that EGFR and integrin signaling events overlap to promote HCMV-induced monocyte motility through actin cytoskeletal rearrangement, although the specific signaling cascades downstream of these receptors regulate distinct cellular molecules. We also note that the requirement of both EGFR and integrin signaling for actin cytoskeletal rearrangement in the process of viral entry into monocytes links the receptor-mediated activation of cellular motility to the viral entry process, as the cytoskeletal events regulating enhanced motility also regulate viral entry [26,45,46]. The EGFR- and integrin-mediated enhanced motility of infected monocytes, when paired with a virus-induced increase in monocyte adhesion molecules and in monocyte transendothelial migration, likely allows the infected monocyte to exit the bloodstream and to enter peripheral organ tissues [22,23]. Furthermore, HCMV-induced activation of EGFR alone results in the prolonged survival of infected monocytes by altering the normal cellular apoptotic machinery through the prolonged expression of Mcl-1, providing the virus with a longer-lived cellular host [22,25]. The enhanced survival of infected monocytes is also potentiated by HCMV-induced differentiation of infected monocytes to naturally long-lived tissue macrophages within the organ tissues [21,24]. Following the differentiation of infected monocytes to macrophages, viral replication can then commence [21,51,52], resulting in progeny virions capable of infecting the necessary surrounding cell types required to promote viral shedding to other hosts [13,21] or capable of infecting myeloid progenitor cells within the bone marrow to establish the latent infection required for lifelong viral persistence within the host [19,28,29,32]. 

These earlier studies highlight the ability of HCMV to alter the biological function of infected monocytes to promote changes that are critical for the outcome of the viral life cycle and for the viral persistence strategy. Furthermore, our studies demonstrate that the HCMV-induced changes in infected monocytes are initiated by cellular surface receptor binding-induced signaling in the cell, in the absence of *de novo* viral gene expression. In fact, newer studies have uncovered that a high degree of regulatory control is utilized by HCMV during infection of monocytes through the utilization of these two separate cellular receptors, EGFR and integrins, to promote a “finely-tuned” monocyte/macrophage cell type that is required for viral spread, replication, and persistence. 

## 3. Precise Regulation of the Monocyte/Macrophage Apoptotic Program Is Required for HCMV-Induced Monocyte-to-Macrophage Differentiation

We have shown that HCMV effectively initiates molecular changes that reprogram infected monocytes to establish a pro-viral phenotype that mediates viral dissemination. We suggest that for the virus to successfully have evolved to infect monocytes and utilize them as “Trojan Horses” for the spread of HCMV to target organs, the virus had to overcome at least two significant biological obstacles. First, HCMV had to adapt to the naturally short lifespan of monocytes of around 48 to 72 hours in circulation [34,35], a scenario that is particularly problematic for HCMV, with its slow DNA replication cycle of several days to weeks *in vivo* [53]. Second, the virus had to adapt to the differentiation barrier, because as stated previously, monocytes are not initially permissive for *de novo* viral gene expression and replication [14,52,53], while their differentiated counterparts, macrophages, are permissive [21]. We have evidence that HCMV navigates these biological hurdles by extending the lifespan of infected monocytes and directing their differentiation into long-lived macrophages, which are capable of supporting viral replication and the production of progeny virions [21,24,25,44]. In this manner, it appears that HCMV has evolved a mechanism to molecularly bridge the survival‑differentiation hurdle in order to generate a long-lived cell type that serves as a reservoir for viral persistence in host organ tissue. 

In order to promote the extended survival of HCMV-infected monocytes, HCMV must escape both the intrinsic programming that causes monocytes to die within 48–72 hours of entering circulation [34,35] and the cellular antiviral pro-apoptotic response [54]. Because monocytes are not productive for viral gene expression and replication, viral immediate early proteins do not participate in the rescue of infected monocytes from various apoptotic mechanisms. Accordingly, the virus appears to have evolved a mechanism whereby it relies on cellular Bcl-2 family anti-apoptotic proteins in order to navigate critical cellular checkpoints and to escape apoptosis. The Bcl-2 family of proteins consists of multiple mitochondrial membrane permeabilization regulators that function to either promote or inhibit apoptosis [55]. Anti-apoptotic Bcl-2 members (Mcl-1, Bcl-2, and Bcl-xL) have been reported to mediate survival of myeloid cells under some conditions [56,57,58,59,60,61]. Our laboratory recently reported that Mcl-1 functions to mediate the early survival of infected monocytes [25]. Monocytes have a biologically limited lifespan of about 48–72 hours in the circulation, during which they must make a cell fate decision to either undergo monocyte-to-macrophage differentiation [24,25] or to fulfill their natural “default” biological programming and undergo apoptosis. Under homeostatic conditions, monocytes exhibit high levels of Mcl-1 upon entry into circulation and upon isolation in our system, followed by a significant reduction in Mcl-1 protein levels over their 48–72 hour lifespan [25]. The decline of pro-survival Mcl‑1 between 48 and 72 hours post infection is consistent with the *in vivo* lifespan of these cells and appears to constitute a potential molecular viability gate, which monocytes must negotiate in order to escape their intrinsic apoptotic fate [25]. Furthermore, this expression pattern suggests that, under homeostatic conditions, Mcl-1 may function as a molecular clock to ensure a controlled population of unstimulated short-lived monocytes [25]. These data are consistent with what is known about Mcl-1 and its anti-apoptotic function in myeloid leukemia cells [62,63,64]. The Mcl-1 gene was originally identified as being over-expressed in a human myeloid leukemia cell line during monocyte-to-macrophage differentiation [62]. Mcl-1 has since been identified as a member of the Bcl-2 gene family that, under homeostatic conditions, serves to regulate two important myeloid cell processes: survival and differentiation [63,65]. Because of its pro-survival function in cells, over-expression of Mcl-1 enhances the survival of cancerous cells and contributes to drug resistance in a variety of cancers [66,67,68,69]. Our data suggest that HCMV usurps the pro-survival function of Mcl-1 to promote the enhanced survival of infected monocytes. In contrast to uninfected monocytes, Mcl-1 levels remain stable through the first 24 hours following HCMV infection; after 24 hours, Mcl-1 levels in infected monocytes decline until expression finally reaches the same levels as those observed in mock-infected cells at 72 hours post infection [25]. By extending the expression of Mcl-1 in HCMV-infected monocytes when compared to their mock-infected counterparts, HCMV infection allows these cells to navigate the cell viability checkpoint at 48 hours post infection. Thus, HCMV appears to upregulate Mcl-1 expression in infected monocytes to overcome the intrinsic apoptotic clock that controls the monocyte population under homeostatic conditions, in order to extend the lifespan of the infected cell [25]. In support, siRNA-mediated knock down of Mcl-1 levels leads to a reversal of the apoptotic resistance observed in HCMV-infected monocytes, confirming that Mcl-1 is responsible for the enhanced survival of monocytes during the initial 24–48 hours following infection [25]. 

Molecularly, survival and differentiation are distinct processes in monocytes that are, nonetheless, intimately linked. Prolonged survival of monocytes is necessary to afford the time required for differentiation to occur; however, an excess of pro-survival signals can block differentiation [70]. Thus, the survival and differentiation programs in monocytes appear to represent a “Goldilocks” phenomenon whereby only precise expression levels of the cellular products involved in both survival and differentiation will result in effective monocyte-to-macrophage differentiation. In fact, myeloid cell differentiation has been shown to require low level expression of multiple “pro-apoptotic” players, particularly the caspases [70]. For example, caspase-3 and caspase-8 play a significant role in monocyte-to-macrophage differentiation induced by stimulation with macrophage colony-stimulating factor (M-CSF): both caspases are required for differentiation to occur [70]. However, the expression of caspase-3 and caspase-8 is lower during differentiation than during apoptosis [70], suggesting a threshold level of caspase expression exists that is necessary for differentiation, while remaining low enough to avoid triggering apoptosis. These studies implicate that the process of monocyte-to-macrophage differentiation may represent, molecularly, an incomplete apoptosis-like program. 

In support, recent work from our laboratory has shown that HCMV controls monocyte-to-macrophage differentiation through the partial activation of caspase-3 [24]. Furthermore, we demonstrated that, prior to the 48-hour cell fate checkpoint, Mcl-1 acts to inhibit the cleavage and activation of caspase-3, which in turn promotes monocyte survival [24]. However, once infected monocytes survive to and through the 48-hour viability gate, the loss of Mcl-1 allows a basal activation of caspase-3 that is required for monocyte-to-macrophage differentiation [24]. Thus, by differentially regulating Mcl-1 expression levels, HCMV walks a fine line between survival and death; between differentiation and non-differentiation, to create the long-lived macrophage that is central to its viral survival/persistence strategy. 

**Figure 1:**
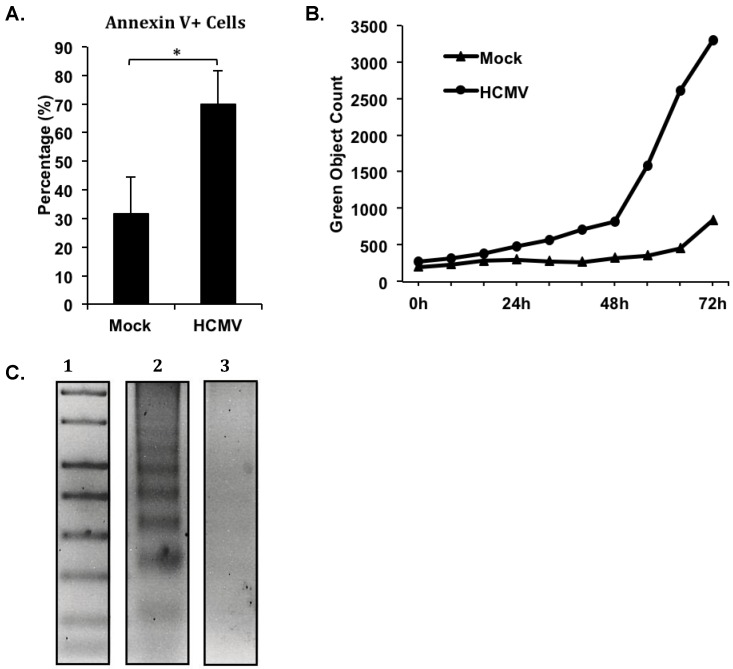
Human cytomegalovirus (HCMV) induces the upregulation of apoptosis-associated markers in the absence of increased apoptosis. Primary human peripheral blood monocytes were isolated as previously described [22,44]. Monocytes were mock- or HCMV-infected (Towne/E p.41 MOI 5) for 72 hours at 37 °C in 5% CO_2_, and kept either in suspension (**A** and **C**) or adherent in a 96 well plate (**B**) in 1% human serum RPMI 1640 (Cellgro). (**A**) At 72 hours post infection, monocytes were harvested, stained with Annexin V-FITC and PI, and analyzed by flow cytometry. The average percentage of Annexin V positive, PI negative cells in each sample from three separate donors is represented. Student’s paired t-test was used to determine statistical significance (* *p* < 0.05). (**B**) At the time of infection, triplicate samples were treated with 5 μM CellPlayer^™^ 96-Well Caspase-3/-7 reagent. Samples were placed in an IncuCyte^™^ Zoom live imaging system at 37 °C and 5% CO_2_. Phase contrast and green fluorescence images of each sample at identical areas were acquired every six hours for 72 hours. The number of green objects detected per image was normalized to the number of cells detected by phase contrast per respective image. The resulting green object count for each sample is represented. (**C**) At 120 hours post infection, fragmented DNA was harvested from samples using a Calbiochem^®^ Suicide Track^™^ DNA Ladder Isolation kit. Isolated DNA was run on a 1.5% agarose/TAE gel at 50 constant volts. The gel was then stained with ethidium bromide and apoptotic DNA was visualized by UV illumination. All samples were run on the same gel under the same conditions; however, a lane containing an irrelevant treatment option was removed for the final figure. Lane 1 contains the 1 kb DNA standard ladder, Lane 2 contains DNA from mock-infected monocytes, and Lane 3 contains DNA from HCMV-infected monocytes.

Our more recent studies investigating the prolonged survival of HCMV-infected monocytes have further illustrated the involvement of a possible incomplete apoptosis-like program in HCMV-induced monocyte-to-macrophage differentiation. During our analysis of a panel of assays to identify apoptotic cell death, we identified that despite the well-characterized enhanced survival of HCMV-infected monocytes [24,25], HCMV-infected cells did not exhibit a decrease in the expression of several apoptosis-associated markers as would be expected, but rather showed an increase in some quantifiable phenomena associated with apoptosis (Figure 1). Flow cytometric analysis of Annexin V and propidium iodide (PI) staining in HCMV-infected *versus* mock-infected monocytes at 72 hours post infection revealed a greater than two fold increase in the percentage of Annexin V positive, PI negative cells in HCMV-infected monocytes (Figure 1A). Cells staining as Annexin V positive, PI negative are prototypically considered to be in the early stages of apoptosis, because Annexin V binds to phosphatidyl serine (PS), an inner membrane component that flips to the outer membrane of cells during apoptosis [71]. However, the presence of PS on the outer membrane of cells is also a natural occurrence during monocyte-to-macrophage differentiation, and PS on the outer membrane is required for the phagocytic function of macrophages [72]. With an already documented increase in cell viability during HCMV infection of monocytes [45] and in the absence of an increase in PI staining, which would represent true cell death; the increase in the number of cells expressing PS (by Annexin V staining) in HCMV-infected monocytes likely represents the larger number of cells undergoing monocyte-to-macrophage differentiation that we have previously documented [21,24]. Furthermore, quantification of fluorescent cells in images captured by an IncuCyte ZOOM^™^ analyzer of HCMV-infected *versus* mock-infected monocytes that were treated with CellPlayer™ 96-well Caspase-3/7 reagent (which fluorescently labels cells containing activated or cleaved caspase-3/7) over a timecourse of 72 hours indicated an increase in the number of cells containing cleaved caspase-3/7 in HCMV‑infected cells compared to mock-infected cells (Figure 1B). We previously showed that HCMV-infected monocytes had increased levels of the full-length 32 kDa and partially cleaved 20‑kDa forms of caspase-3, while showing similar levels of the fully-cleaved 17 kDa form of caspase‑3, when compared to mock-infected monocytes by three days post infection [24]. Additionally, we have observed an increase in caspase-7 expression and cleavage in infected monocytes 3–5 days post infection [73]. Although the activation of caspase-3/7 is typically indicative of an apoptotic process in cells, our previous data showed that caspase-3 activity was required for HCMV‑induced monocyte-to-macrophage differentiation [24]; however, we have not yet addressed a potential role for caspase-7 in the differentiation of infected monocytes to macrophages. With a defined role for caspase-3 activation in myeloid cell differentiation [24,64] and the recently documented role for caspase-7 in the differentiation of dental hard tissue producing cells, such as odontoblasts and ameloblasts [74], we suggest that the increase in the number of cells expressing activated caspase-3 and -7 in HCMV-infected monocytes, again reflects a greater number of cells undergoing monocyte-to-macrophage differentiation, rather than apoptosis. To verify that HCMV‑infected monocytes exhibited lower amounts of apoptosis than their uninfected counterparts despite the measurable increase in PS expression and caspase-3/-7 activation, we performed a DNA laddering assay to detect the presence of fragmented DNA (a hallmark of complete apoptosis) at 72 hours (data not shown) and 120 hours post infection (a time that we could identify any cellular apoptosis that may potentially be initiated at 72 hours) (Figure 1C). We observed a small amount of DNA laddering in HCMV-infected monocytes (Figure 1C, Lane 3), indicating that some HCMV-infected cells had undergone apoptosis. However, we identified that uninfected monocytes (Figure 1C, Lane 2) had an increased amount of DNA laddering when compared to HCMV-infected monocytes (Figure 1C, Lane 3), indicating the presence of increased apoptosis in uninfected monocytes. Therefore, it appears that although multiple biological changes occur during HCMV infection of monocytes that have been linked to the initiation of an apoptotic cascade, full apoptosis does not occur in the majority of HCMV‑infected monocytes. We argue that the process of macrophage differentiation likely requires the specific regulation of a partial apoptotic-like cascade (involving multiple steps including the activation of caspases and the surface expression of PS) that drives the myeloid differentiation process to a “point of no return” for the macrophage, but that does not result in a full apoptotic response that would result in cell death.

## 4. HCMV-Induced Monocyte-to-Macrophage Differentiation Is Required for Viral Replication and for Long-Term Persistent Infection

Infection of monocytes by HCMV was initially thought to represent a non-productive and abortive infection for the virus because monocytes do not support viral replication [8,12,36]. However, as previously discussed, HCMV-infected monocytes are clearly implicated in the overall dissemination of HCMV within the infected host [8,9,10,11,12,17,18]. The circulating surveillance function of monocytes within the bloodstream makes this highly motile cell type ideal for delivery of the virus to multiple organ tissues, but in the absence of viral replication, infection of monocytes by HCMV would appear to serve no productive function for the overall viral persistence strategy. On the other hand, viral replication has been shown to occur in monocyte-derived macrophages [13,14,15,16,75] and in macrophages from infected individuals [13,76]. However, differentiated macrophages are not found within the bloodstream and can, therefore, not play a predominant role in the hematogenous dissemination strategy of HCMV during primary infection. Our laboratory demonstrated that HCMV is able to promote the transendothelial migration of infected monocytes to facilitate their exit out of the bloodstream and entry into the surrounding tissues [21,22]. Studies of a mouse model of CMV infection support the extravasation of infected monocytes out of the bloodstream and their movement into organ tissues [77]. In this model, a primarily mononuclear cell-associated CMV viremia was identified in the infected mice; that was then followed by a dissemination of the virus to the liver and spleen [77]. Furthermore, we showed that primary infection of monocytes by HCMV drives the differentiation of infected monocytes into macrophages that can then support viral replication and the release of infectious virions [21,24,78,79]. Although infected monocytes appear to be driven towards differentiation into macrophages within hours following infection [78,79], the complete productive monocyte-to-macrophage differentiation process appears to take up to two weeks post infection [21]. HCMV-infected monocytes exhibit gene expression changes that are associated with macrophage differentiation as early as four hours post infection [78,79] and have an upregulation of macrophage markers, such as CD68, as early as 24 hours post infection [21]. HCMV-infected monocytes gain an increase in macrophage-associated phagocytosis function by three days post infection [21]. The infected monocyte/macrophage begins to express the macrophage differentiation marker, HLA-DR, in low levels by four days post infection, with increasing expression levels that peak around two weeks post infection [21]. The HCMV immediate early (IE) gene products, IE1 and IE2, and the late gene product, gH, can then be detected within these fully differentiated macrophages between 2–3 weeks post infection, which coincides with the release of infectious virus into the macrophage supernatant [21,46]. Together, these data demonstrate that primary infected peripheral blood monocytes not only serve as viral carriers for hematogenous dissemination, but that they also serve as sources of viral persistence through their production of progeny virions.

The release of newly produced infectious virions by HCMV-infected monocytes that have undergone differentiation into tissue macrophages would allow the virus to establish a long-term infection within peripheral organ tissue. It is likely that this viral persistence in macrophages links HCMV infection to the overt organ pathology observed in immunocompromised hosts. Newly released infectious virions within various host organs would then be able to infect additional recruited myeloid cells and nearby epithelial cells at sites of bodily fluid excretion in order to facilitate chronic viral shedding and passage to new hosts [20,22]. In support, following the initiation of viral gene expression around two weeks post infection, we detect the release of infectious HCMV into the cellular supernatant at around three weeks post infection, which we showed continues through five weeks post infection [21]. Furthermore, we now have data indicating that HCMV-induced differentiation of infected monocytes results in a long-lived macrophage phenotype that becomes a long-term persistent source for the production of infectious HCMV (Table 1). In fact, HCMV-infected monocyte-derived macrophages secreted infectious virus into the cellular supernatant for at least 16 weeks post infection, albeit at very low levels (Table 1). Therefore, when paired with our previous data indicating that HCMV drives the motility and transendothelial migration [21,26,45] and the prolonged survival [25] and differentiation of infected monocytes [21,22,24], these data suggest that primary infected peripheral blood monocytes serve a critical role in the overall long-term persistence strategy of HCMV. Through the induction of these highly specific biological changes in infected monocytes, HCMV promotes the establishment of a long-lived macrophage phenotype that supports the chronic production of infectious virus, likely contributing to continuous shedding of the virus for months after initial infection. Clinical data support that chronic shedding of the virus following primary infection is a common occurrence, particularly among children in daycare settings, who have been shown to shed the virus for up to 23 months [80]. Furthermore, because monocytes/macrophages are the primary cell type involved in carrying reactivated HCMV, the replication of reactivated HCMV within these cells may contribute to the observed chronic shedding of HCMV in the breast milk of postpartum mothers [81]. Therefore, the replication of HCMV within differentiated monocytes/macrophages likely serves as a critical component for the long-term production and shedding of HCMV in both primary and reactivated infections.

**Table 1:** Long-term release of HCMV from infected monocytes/macrophages. Monocytes were mock- or HCMV-infected (Towne/E MOI 5) and then cultured for 16 weeks. At the indicated times post infection, cell supernatants were collected and added to fibroblasts. The presence or absence of detectable IE1 expression was determined by immunohistochemistry staining using an anti-IE1 antibody (Millipore MAB810), Histostain-Plus Kit (Invitrogen cat. 859043), and AEC Substrate Kit (Invitrogen cat. 00-2007) at 24 hours post infection, as an indication for productive virus release by the long-term infected cells. “-” represents the absence of released virus and the numbers represent the calculated plaque forming units per 500 macrophages (averaged from two donors).

Treatment	2 weeks	4 weeks	8 weeks	12 weeks	16 weeks
Mock	-	-	-	-	-
HCMV	-	20	26	30	10

## 5. HCMV Reprograms the Polarization of Infected Monocytes towards a Unique Macrophage Phenotype in Order to Promote Viral Replication and Persistence

Recent advances in macrophage biology research have uncovered that the differentiation of monocytes into macrophages can result in a wide spectrum of resulting phenotypes that have a variety of biological functions [82,83,84,85]. These macrophage phenotypes have been categorized broadly into “M1”, or classically-activated, pro-inflammatory macrophages, and “M2”, or alternatively-activated, anti-inflammatory macrophages, although the phenotypes classified within these categories are broadly pleotropic [82,83,84,85]. Differentiation towards an M1 macrophage phenotype typically occurs following activation by interferon-γ (IFN-γ), granulocyte-macrophage colony-stimulating factor (GM-CSF), tumor necrosis factor (TNF), or lipopolysaccharide (LPS), resulting in a host defense or tumor resistance phenotype [83,84,85]. M1 macrophages are characterized by their secretion of large amounts of proinflammatory cytokines, including interleukin (IL)-6, IL-12, IL-23, and TNF [84,86,87]. On the other hand, differentiation towards an M2 macrophage phenotype typically occurs following activation by IL-4, IL-10, IL-13, or M-CSF, resulting in a regulatory, anti-parasitic, wound healing, or tumor promotion phenotype [83,84,85]. M2 macrophages are characterized by the secretion of very low levels of inflammatory cytokines and the production of high amounts of IL-10 [84,86,87]. Moreover, M2 macrophages have been further classified into three subcategories (M2a, M2b, and M2c), which differ by stimulating cytokines, upregulated macrophage markers, cytokine production, and distinct biological function [84]. For example, M2a macrophages are stimulated by IL-4 and IL-13, express scavenger receptors on their surfaces, and function primarily in anti-parasitic activity or allergic inflammation; M2b macrophages are stimulated by Toll-like receptors or IL-1 receptors, express CD86, secrete TNF, IL-1, and IL-6, and are involved in the regulation of immune responses; and M2c macrophages are stimulated by IL-10, secrete TGF-β, and are involved in tissue remodeling [84]. Therefore, the specific activating ligand involved in the stimulation of monocytes can greatly influence the overall differentiation and polarization of monocytes to macrophages. 

Regardless of stimuli, the process of monocyte-to-macrophage differentiation results in a series of cellular phenotypes that have some degree of shared functional and/or cytokine expression traits along with distinct characteristics. Thus, due to the wide range of potential differentiation-inducing ligands, the overall resulting macrophage cell type under various stimuli represents a diversity of potential cell types. Our laboratory was among the first to demonstrate that a virally-induced monocyte-to-macrophage differentiation process occurs and that it is likely required for viral replication and long‑term viral persistence [21,24,78,79]. Because macrophages can be polarized to function generally as either pro-inflammatory/anti-microbial or anti-inflammatory/pro-tumorigenic, the specific type of macrophage polarization that results during HCMV infection of monocytes would have significant implications for viral dissemination and persistence. Our laboratory’s data indicate that HCMV induces the pro-inflammatory activation of infected monocytes, which we argue is critical for viral spread within the host [21,23,45]. However, the induction of a pro-inflammatory or M1 phenotype typically results in an anti-microbial functional that is characteristic of a host immune response [83,84,88], which would likely be detrimental to the viral life cycle. Nonetheless, because of the plasticity of macrophage differentiation along with our data that the stimulation of macrophage differentiation by LPS results in a macrophage phenotype that is distinct from HCMV-induced macrophage differentiation [21], we suggest that HCMV utilizes this plasticity to regulate the polarization of infected monocytes/macrophages towards a distinct phenotype that ensures successful viral survival and persistence. One mechanism to test this notion of a distinct cell type is global transcriptome microarray analyses. We used this global transcriptome approach paired with an analysis of secreted chemokine analysis to decipher the distinct polarization of HCMV-infected monocytes/macrophages [78,79]. These studies identified that HCMV-infected monocytes exhibit a unique reprogramming of their differentiation and polarization in which both M1- and M2-associated genes are expressed and chemokines are secreted [78,79]. Specifically, we identified that subsets of M1-associated gene transcripts (65%) and M2-associated gene transcripts (4%) were upregulated during HCMV infection of monocytes, and subsets of M1-associated chemokines (44%) and M2-associated chemokines (33%) were more abundant in infected monocytes. These data implicate that HCMV-infected monocytes/macrophages fall somewhere along the macrophage polarization continuum to resemble a more “M1-like” pro-inflammatory phenotype [78,79]. We propose that HCMV specifically modulates the polarization of infected monocytes/macrophages to induce an activated, mostly M1-like phenotype in order to promote the pro-inflammatory changes (such as adhesion, motility, and survival) that are required for effective viral dissemination while dampening a potential anti-viral immune response by promoting the upregulation of key M2-associated genes (such as IL-10 and CCL18). 

**Figure 2:**
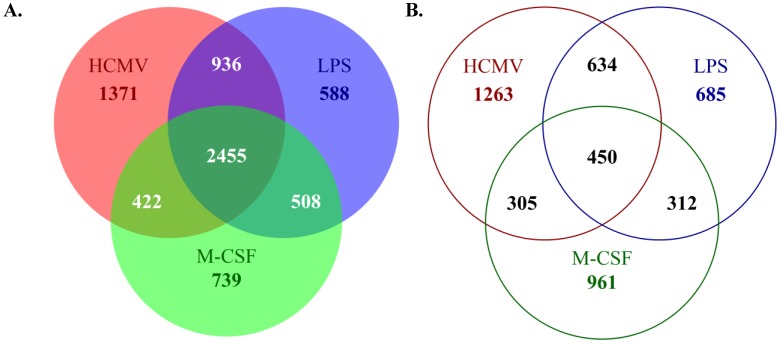
HCMV infection of monocytes induces a unique subset of gene expression changes when compared to monocytes polarized towards an M1 or M2 phenotype. Monocytes were mock- or HCMV-infected (Towne/E p.41 MOI 5), or treated with LPS (100 ng/mL) or M-CSF (100 ng/mL) for two weeks at 37 °C and 5% CO_2_ under adherent conditions. At two weeks, RNA was harvested from each sample. Harvested RNA was used to perform microarray analysis on Affymetrix U133 Plus 2.0 genechips. Probe sets to represent individual genes were selected by using JetSet [89]. The genes that were upregulated (**A**) or downregulated (**B**) (≥2 fold change when compared to mock-infected monocytes) were compared between treatments and the number of unique or shared genes among samples is represented.

To expand our microarray analyses, we have focused on the identification of specific gene expression changes that occur during monocyte-to-macrophage differentiation and polarization under various stimuli. These studies were designed to allow us to determine specific genes that are key to the overall differentiation of monocytes to macrophages, regardless of stimuli, and moreover, to identify the specific subsets of gene expression changes that are shared among HCMV-infected and M1 or M2 polarized macrophages. We hoped from these studies to gain a better understanding of the similarities and differences of HCMV-induced macrophage polarization *versus* traditional M1 or M2 polarization. To address this question, monocytes were mock- or HCMV-infected, or treated with LPS (to induce M1 polarization) or M-CSF (to induce M2 polarization), for two weeks*.* At two weeks, RNA was harvested for microarray analysis. These new microarray analyses have uncovered the differences in the overall gene expression profile and the changes that occur during the differentiation of HCMV-infected *versus* LPS-treated (M1) *versus* M-CSF-treated (M2) macrophages, when compared to mock-infected monocytes/macrophages (Figure 2A,B). Our data indicate that the general ligand-induced monocyte-to-macrophage differentiation process includes a subset of gene expression changes that is present regardless of stimulating ligand or polarization (upregulation of 2,455 genes; downregulation of 450 genes) (Figure 2A,B). That is, there is a core set of likely “pan” macrophage genes induced and/or reduced. However, the differentiation of monocytes towards an M1 or M2 macrophage phenotype also resulted in a large pool of distinct gene expression profiles. That is, we observed a large number of gene expression changes that appear unique to the specific macrophage polarization phenotype (upregulation of 739 M-CSF-specific genes and 588 LPS-specific genes; downregulation of 961 M-CSF-specific genes and 685 LPS-specific genes) (Figure 2A,B). Furthermore, these analyses revealed that HCMV-induced monocyte-to-macrophage differentiation results in subsets of gene expression changes that are shared with either M1 or M2 macrophages, with more genes shared with the M1 macrophage polarization phenotype (upregulation of 936 LPS-shared genes; downregulation of 634 LPS-shared genes) than the M2 macrophage phenotype (upregulation of 422 M-CSF-shared genes; downregulation of 305 M-CSF-shared genes) (Figure 2A,B). We also observed a distinct subset of gene expression changes in infected monocytes/macrophages that were not observed in either M1 or M2 polarized macrophages (upregulation of 1,371 HCMV-specific genes; downregulation of 1,263 HCMV-specific genes) (Figure 2A,B), suggesting that the HCMV-induced differentiation program of infected monocytes truly represents a unique differentiation phenotype. 

**Figure 3:**
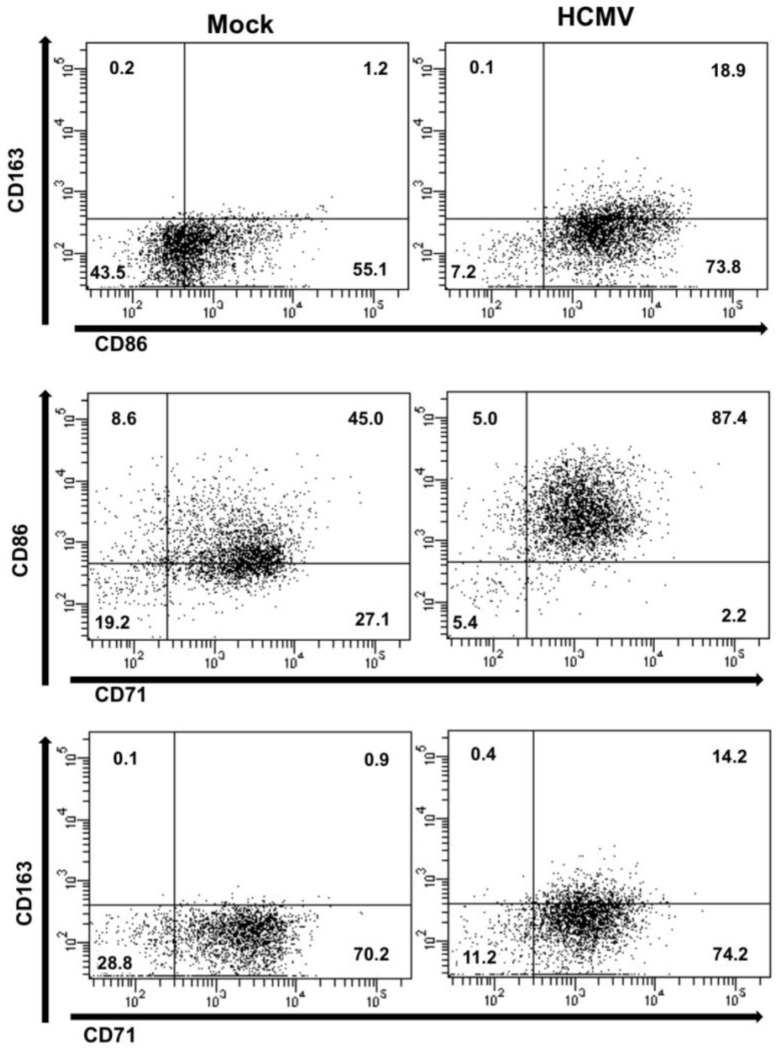
HCMV infection of monocytes induces a macrophage polarization phenotype that is distinct from M1 or M2 polarization. Monocytes were mock- or HCMV-infected (Towne/E p.41 MOI 5) at 37 °C and 5% CO_2_ for five days. Monocytes/macrophages were washed with PBS, blocked in 5% fetal bovine serum and stained with Alexa fluor-conjugated antibodies specific for CD14 (a myeloid specific marker), CD71 (a macrophage specific marker), CD86 (an M1 macrophage associated marker), or CD163 (an M2 macrophage associated marker). Cells were then analyzed by flow cytometry and gated by forward and side scatter and for CD14 positive cells. Scatter quadrant plots were generated indicating the number of cells expressing the various staining profiles.

In support of our gene array analyses, flow cytometric analysis revealed an increase in the expression of both M1- and M2-associated macrophage markers in HCMV-infected monocytes when compared to mock-infected monocytes at five days post infection (Figure 3). Of note—although uninfected monocytes are programmed to die between 48–72 hours in circulation, a certain percentage of cells (~40%) receive enough stimuli in our *in vitro* culture system to survive and undergo a small degree of differentiation. However, when compared to the percentage of cells found in HCMV-infected monocytes (>80% survival), it is clear that infection serves as both a survival and a differentiation stimuli. Analysis of co-staining with CD71 (a general macrophage marker), CD86 (a cell surface marker that is typically expressed on M1 macrophages), and CD163 (a cell surface marker that is typically expressed on M2 macrophages) revealed that HCMV infection of monocytes induces the upregulation of specific macrophage markers, resulting in a larger number of cells within the doublet staining macrophage subsets. In addition, the macrophage marker expression profile of HCMV-infected monocytes was distinct from that seen with M-CSF- or GM-CSF-treated monocytes, suggesting that various stimulating ligands induce distinct macrophage phenotype populations (data not shown). Furthermore, analyses of the expression of select M1- and M2-associated genes in monocytes/macrophages at 24 and 72 hours following treatment with M-CSF, GM-CSF or LPS or infection with HCMV supported the finding that HCMV infection of monocytes/macrophages results in a distinct polarization profile that shares some characteristics with M1 and/or M2 macrophages (data not shown). However, further studies involving additional donors are required to effectively decipher gene profile changes that occur during macrophage polarization between 24 and 72 hours. Taken together, these data demonstrate that HCMV-infection of monocytes/macrophages drives their differentiation/polarization towards a macrophage phenotype that shares some characteristics with M1‑polarized macrophages and M2-polarized macrophages, but that, overall, represents a distinct polarization phenotype; that is, the specific HCMV-induced polarization phenotype does not fall into the category of strictly “M1” or “M2”, but exists somewhere along the M1/M2 continuum, skewed towards a more M1-like phenotype.

Overall, we propose that this unique macrophage polarization phenotype uncovered by our studies reflects the manipulation of the differentiation and polarization program of infected monocytes by HCMV to promote a macrophage phenotype that is required for the overall persistence strategy of the virus. We predict that the early pro-inflammatory phenotype of HCMV-infected monocytes/macrophages regulates the motility and migration of the infected cells in order to promote their exit out of the bloodstream, and it helps to trigger the pro-survival signals necessary to overcome the cell’s intrinsic apoptotic program (inflammatory activation is known to induce prolonged monocyte survival [90]). However, because some inflammatory cytokines have also been linked to apoptosis in *Mycobacterium-*infected monocytes [90], the expression of some M2-associated anti-inflammatory genes in HCMV‑infected monocytes likely helps to dampen the cell’s intrinsic apoptotic host response to infection. Through the specific regulation of the differentiation and polarization of infected monocytes/macrophages, we argue that HCMV promotes the specific cellular environment that is required for viral replication and overall persistence. This notion is supported by studies involving the infection of already differentiated M1 or M2 macrophages by HCMV, which indicate that although both macrophage phenotypes are susceptible to HCMV infection, HCMV is able to infect a higher percentage of M2 macrophages than M1 macrophages, indicating that M2 macrophages are more susceptible to HCMV infection [91,92]. These studies also indicated that HCMV induced the secretion of pro-inflammatory cytokines and chemokines in both M1 and M2 macrophages following infection, but that infected M2 macrophages released a higher amount of infectious virus [93]. These data suggest that HCMV not only induces the polarization of differentiating monocytes, but also alters the polarization profile of already differentiated macrophages by inducing the expression of pro‑inflammatory molecules. Furthermore, these data suggest that the expression of specific M2‑associated molecules confers some type of advantage for the virus during infection, indicating that the specific macrophage polarization phenotype in infected cells may determine the overall outcome of HCMV replication and gene expression. In support, it was shown that the specific phenotype of differentiated macrophages resulting from infected monocytes strictly affected HCMV replication and the production of infectious virus from infected monocytes/macrophages, as the absence of IFN-γ and IL-2 nearly abolished viral replication [52]. Moreover, IFN-γ and TNF-α were shown to be required for the differentiation of HCMV-permissive monocyte-derived macrophages, and the presence of these cytokines during the differentiation process conferred HCMV resistance to these antiviral cytokines [93]. Taken together, these studies illustrate the requirement of a very distinct macrophage phenotype, expressing specific proinflammatory (M1-associated) cytokines and/or cellular factors (such as IFN-γ and TNF-α) and certain anti-inflammatory (M2-associated) cytokines and/or cellular factors (such as IL-10), for the establishment of the ideal tissue environment for effective HCMV replication within monocyte-derived macrophages. Therefore, we propose that the “fine-tuning” of the infected macrophage polarization phenotype likely produces a cellular environment that expresses all of the cytokines and cellular factors that are required for the hematogenous spread of HCMV and for the initiation of HCMV replication in macrophages, while avoiding certain detrimental outcomes like a robust antiviral immune response. Specifically, we predict that the expression of M1-associated cytokines likely promotes the required biological changes for enhanced motility, transendothelial migration, and survival to promote viral spread and replication and to help provide resistance to any later production of these pro-inflammatory cytokines, while the expression of M2-associated cytokines promotes an environment that is better-suited for viral replication and that helps to dampen immune detection of the virus.

## 6. Discussion

The hematogenous dissemination strategy of HCMV allows the virus to spread throughout the entire body of the host and to gain access to nearly every organ tissue. This systemic spread is critical for the establishment of viral latency within the bone marrow and the life-long persistent infection that is associated with HCMV infection [19,28,29]. Because peripheral blood monocytes play an immune surveillance role within the host and can, therefore, easily transition out of the bloodstream to enter into peripheral organ tissues, monocytes represent ideal candidates to serve as viral carriers for the spread of HCMV during infection. Furthermore, the clinical disease manifestations associated with HCMV infection are thought to result from the infiltration of infected monocytes during the hematogenous spread of the virus [94]. However, because peripheral blood monocytes have a short lifespan [34,35] and do not support *de novo* viral gene expression or replication until differentiation into macrophages [36], HCMV must first overcome these biological hurdles in order to utilize monocytes for viral spread.

**Figure 4:**
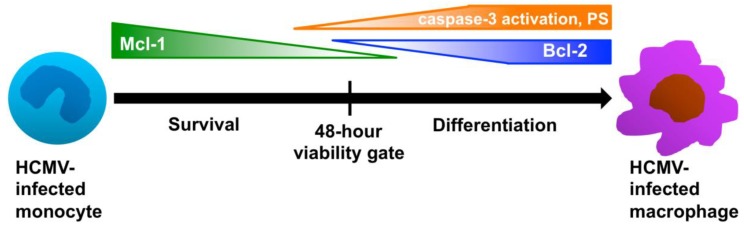
HCMV regulates the distinct processes of survival and differentiation during infection through the precise expression of specific pro- and anti-apoptotic molecules. Our data have shown that HCMV infection induces the enhanced expression of Mcl-1 in infected monocytes in order to mediate their prolonged survival through a 48-hour viability checkpoint, a time in which uninfected monocytes are programmed to die [24,25]. Mcl-1 levels gradually decrease through 48 hours after infection, before becoming undetectable. Following 48 hours, HCMV induces the enhanced expression of Bcl-2 and phosphatidyl serine (PS) membrane expression and an increase in the partial activation of caspase-3 in infected monocytes. The expression of low levels of Bcl-2 helps to support the survival of infected monocytes after the 48-hour viability gate, while precise regulation of the membrane expression of PS and the partial activation of caspase-3 help to drive the differentiation of infected monocytes towards replication-permissive macrophages. Overall, through the tight regulation of pro- and anti-apoptotic molecules, HCMV is able to overcome critical biological hurdles, such as the short lifespan of monocytes and the limit of *de novo* viral gene expression and/or replication in monocytes.

Our laboratory has demonstrated that the binding of HCMV glycoproteins to EGFR and the β_1_ and β_3_ integrins on the surface of monocytes triggers the activation of multiple signaling cascades that mediate the pro-inflammatory activation of infected monocytes [26,45]. We propose that this pro‑inflammatory monocyte activation is required for the induction of multiple monocyte biological changes that are critical for viral spread and persistence, including an enhanced motility and transendothelial migration, which help to facilitate the extravasation of infected monocytes out of the bloodstream and into the surrounding tissues [21,22,23,45]. However, due to the natural short lifespan of these cells, in the absence of a viral-induced pro-survival signal, infected monocytes would likely represent an “end-point-cellular-host” for the virus. Our data have shown that HCMV promotes the extended survival of infected monocytes through the prolonged expression of the cellular anti‑apoptotic molecule, Mcl-1 early after infection, which promotes the survival of infected monocytes through a 48-hour viability checkpoint, a time at which uninfected monocytes appear to be programmed to die by apoptosis [25] (Figure 4). Although the prolonged survival of infected monocytes is critical for providing a longer-lived cellular host for the virus, in order for HCMV replication to occur, differentiation of infected monocytes into long-lived macrophages is also required [8,13,14,15,95] (Table 1). Further complicating this scenario, cellular differentiation can by blocked by an abundance of pro-survival signaling [70], suggesting that the survival and differentiation programs in infected monocytes must be intimately linked and tightly co-regulated during HCMV infection in order for viral replication to occur. That is, the virus must regulate the precise expression of pro‑survival signals in order to navigate through critical cell fate decision checkpoints to promote survival, while simultaneously ensuring that differentiation also appropriately commences in the presence of these pro-survival signals. Based on our studies, we have developed a model describing this link between HCMV-induced monocyte survival and differentiation. Early after infection, following the extravasation of infected monocytes into the surrounding tissues, the primary hurdle for HCMV is the “ticking clock” of monocyte pre-programmed cellular death around a 48-hour viability gate (Figure 4). Therefore, HCMV regulates the prolonged expression of Mcl-1 to drive the survival of the infected cell through this 48-hour viability gate (Figure 4). Following the successful navigation through this cell survival “crisis point”, the primary hurdle for HCMV becomes the inability to initiate viral gene expression and replication, thereby necessitating a switch from a primarily cell survival program to a primarily cellular differentiation program (Figure 4). During the cellular differentiation phase of infection, Mcl-1 expression levels are depleted to allow for differentiation, while a low level increase in other pro-survival signals (including Bcl-2 expression) is observed, which we believe allows the infected cell to maintain survival throughout the differentiation process without directly interfering with the differentiation process (Figure 4).

In an effort to identify the molecular mechanisms for how HCMV effectively regulates the distinct processes of survival and differentiation during infection, we initiated studies to molecularly define the players involved in driving the monocyte-to-macrophage differentiation process that may also be linked to alterations in the cell survival program. We have shown that Mcl-1 expression early after infection serves to inhibit the apoptosis of infected cells through the inhibition of caspase-3 cleavage/activation, prior to the 48-hour viability gate (Figure 4) [24]. Furthermore, we showed that once Mcl-1 levels are depleted after the 48-hour viability checkpoint, HCMV utilizes the partial activation of caspase-3, a typically pro-apoptotic molecule that has also been shown to regulate macrophage differentiation [64], in order to drive differentiation of infected monocytes (Figure 4) [24]. Our new studies confirm an increase in the activation of caspases during HCMV infection of monocytes (Figure 1B) and indicate that HCMV infection also induces the expression of PS on the surface of infected monocytes (Figure 1A). Although these findings are typically associated with enhanced cellular apoptosis, we nonetheless observe a prolonged survival [25] and a lower amount of DNA laddering in infected monocytes (Figure 1C), indicating that HCMV promotes resistance to apoptosis in infected monocytes. Moreover, both the activation of caspases [24,64,70,74] and the expression of PS [96] have been shown to occur during the differentiation of monocytes to macrophages. Therefore, we argue that following the successful navigation of infected monocytes through the critical 48-hour cell fate decision checkpoint (which is mediated by the increased expression of cell survival signals, including Mcl-1 [25]), HCMV then initiates the expression of a series of “pro-apoptotic” signals in precise quantities that help to drive the differentiation of infected monocytes to macrophages, but that does not fully activate the apoptotic response (Figure 4). Furthermore, the expression of PS has recently been found to have an anti-inflammatory effect by inducing the expression of anti-inflammatory cytokines that can counteract the effects of pro‑inflammatory cytokines within inflamed tissues [97,98,99,100]. These data suggest that the surface expression of PS in infected monocytes may not only indicate the differentiation of infected monocytes into macrophages, but may also aid in the distinct polarization of infected monocytes/macrophages by enhancing the expression of anti-inflammatory, or M2-associated cytokines to counteract any potential negative or anti-viral effects of pro-inflammatory, or M1-associated cytokines that are expressed during infection. 

## 7. Conclusions

**Figure 5:**
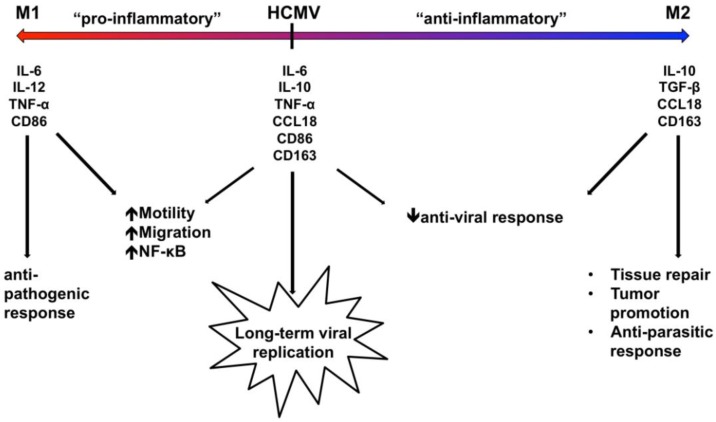
HCMV polarizes infected monocytes/macrophages towards a distinct phenotype possessing aspects of both M1 and M2 macrophages in order to promote viral spread and replication. Our current data indicate that HCMV induces the simultaneous expression of M1-associated molecules (IL-6, TNF-α, CD86) and M2-associated molecules (IL-10 and CD163), suggesting that HCMV-infected macrophages fall somewhere along the macrophage polarization continuum between an M1 and M2 phenotype (with a skewing towards a more M1-like phenotype) with an expression of more M1-associated genes and chemokines than M2-associated genes and chemokines [78,79]. We propose that through tight regulation of the expression of specific M1 (pro-inflammatory) and M2 (anti‑inflammatory) macrophage markers and chemokines, HCMV is able to utilize key pro-viral aspects of both polarization phenotypes, while simultaneously avoiding potential detrimental anti-viral effects. Specifically, HCMV likely utilizes M1-associated macrophage markers and chemokines to promote the pro-inflammatory activation of infected monocytes/macrophages to ensure proper cellular motility, migration, and monocyte recruitment, while utilizing M2-associated macrophage markers and chemokines to dampen the tissue-damaging effects of the pro-inflammatory response and any potential anti-viral responses. Overall, through the precise regulation of macrophage polarization, HCMV likely induces an ideal cellular environment that can support effective long-term viral replication, which ensures the overall success of the viral spread and persistence strategy.

Although the precise mechanisms for how HCMV directs the polarization of infected monocytes/macrophages remain undetermined, our data clearly indicate that HCMV serves as a unique ligand that induces the differentiation and polarization of infected monocytes/macrophages towards a distinct phenotype that shares common characteristics with both M1 and M2 polarized macrophages [78,79] (Figure 2 and Figure 3). In this manner, HCMV appears to specifically regulate the polarization of infected monocytes/macrophages in order to achieve an effective balance between pro‑inflammatory and anti-inflammatory signals, which may establish the cellular environment that is conducive for dissemination and persistence of HCMV (Figure 5). Furthermore, we suggest that the specific polarization of HCMV-infected monocytes into distinct macrophages allows the virus to reprogram its cellular environment to achieve effective viral replication within a now naturally long‑lived tissue macrophage (Figure 5). Combined with our polarization data, our identification of the ability of infected monocytes/macrophages to serve as long-term sources of productive viral release (Table 1) suggests that HCMV specifically manipulates monocyte cell biology to utilize this cell type not only as a vessel for successful dissemination, but to also promote long-term persistence within infected tissues. Furthermore, these studies strengthen previous studies indicating that infected macrophages do, in fact, serve as a critical cellular component in the production of HCMV within tissues, tying infected monocytes/macrophages to viral spread, persistence, and pathogenesis within organs [13,101]. Overall, it seems that in order to successfully reach the point of viral replication, HCMV must orchestrate the precise expression of multiple pro-survival and pro-apoptosis signals, as well as pro‑inflammatory and anti-inflammatory mediators, to achieve optimal “Goldilocks” expression levels of these critical cellular signals in infected monocytes/macrophages. Furthermore, as evidenced by the partial activation of caspase-3 and the extracellular membrane-associated expression of PS on infected monocytes/macrophages, HCMV has likely evolved to utilize multiple molecular mechanisms that link the survival and differentiation/polarization programs of infected cells.
